# 异基因造血干细胞移植治疗≤50岁高危多发性骨髓瘤患者14例临床分析

**DOI:** 10.3760/cma.j.cn121090-20230928-00148

**Published:** 2024-01

**Authors:** 攀 潘, 佳丽 王, 卫华 翟, 巧玲 马, 栋林 杨, 四洲 冯, 明哲 韩, 爱明 庞, 尔烈 姜

**Affiliations:** 1 中国医学科学院血液病医院（中国医学科学院血液学研究所），血液与健康全国重点实验室，国家血液系统疾病临床医学研究中心，细胞生态海河实验室，天津 300020 State Key Laboratory of Experimental Hematlogy, National Clinical Research Center for Blood Diseases, Haihe Laboratory of Cell Ecosystem, Insitute of Hematology & Blood Disarses Hospital, Chinese Academy of Medical Sciences & Peking Union Medical College, Tianjin 300020, China; 2 天津医学健康研究院，天津 301600 Tianjin Institutes of Health Science, Tianjin 301600, China

**Keywords:** 异基因造血干细胞移植, 多发性骨髓瘤, 高危, Allogeneic hematopoietic stem cell transplantation, Multiple myeloma, High risk

## Abstract

**目的:**

观察异基因造血干细胞移植（allo-HSCT）对年轻（≤50岁）高危多发性骨髓瘤（HRMM）患者的疗效。

**方法:**

纳入2016年11月至2022年11月期间于中国医学科学院血液病医院造血干细胞移植中心接受allo-HSCT的14例具有高危细胞遗传学改变或高危疾病生物学因素的年轻（≤50岁）HRMM患者，对其临床资料进行回顾性分析。

**结果:**

14例患者中，男7例，女7例，移植时中位年龄为39.5（31～50）岁。患者移植前中位治疗线数为2（1～6）线，移植前6例患者未达完全缓解（CR），5例患者微小残留病（MRD）阳性。14例患者均获得造血重建，中性粒细胞、血小板中位植入时间分别为11（10～14）d、13（9～103）d。5例患者发生Ⅱ～Ⅳ度急性移植物抗宿主病（GVHD），2例患者发生中重度慢性GVHD。移植后3个月疗效评估，所有患者均为严格意义的完全缓解（sCR）。移植后中位随访时间为18.9（4.1～72.5）个月，移植后2年移植相关死亡率为7.1％（95％ *CI* 0％～21.1％），总生存率为92.9％（95％ *CI* 80.3％～100.0％）；移植后1、2年无进展生存率分别为92.9％（95％*CI* 80.3％～100.0％）、66.0％（95％*CI* 39.4％～100.0％），2年累积复发率为28.9％（95％*CI* 0％～56.7％）。

**结论:**

年轻HRMM患者在诱导治疗后桥接allo-HSCT可进一步提高疗效。

## Abstract (en)

**Objective:**

To evaluate the efficacy of allogeneic hematopoietic stem cell transplantation（allo-HSCT）in young patients with high-risk multiple myeloma（HRMM）and analyzed the factors affecting patient prognosis.

**Methods:**

In this retrospective study, we analyzed the clinical data of 14 patients with HRMM with cytogenetic abnormalities or high-risk biological factors who underwent allo-HSCT at the Hematopoietic Stem Cell Transplantation Center of the Institute of Hematology & Blood Diseases Hospital between November 2016 and November 2022.

**Results:**

There were seven males and seven females included in the study, with a median age of 39.5（31–50）years at the time of allo-HSCT. The median number of treatment lines before transplantation was 2（1–6）. Before allo-HSCT, 42.9%（6/14）of the patients did not achieve complete remission, while 35.7%（5/14）of the patients achieved measurable residual disease positivity. After transplantation, all patients were evaluated for their treatment response, and the overall response rate was 100%（14/14）. All 14 patients successfully underwent allo-HSCT, with median engraftment times for neutrophils and platelets of 11（10–14）days and 13（9–103）days, respectively. Acute grade Ⅱ–Ⅳ graft-versus-host disease（GVHD）occurred in five patients（35.7%）, and two patients（14.3%）developed moderate-to-severe chronic GVHD. The median follow-up time after allo-HSCT was 18.93（4.10–72.53）months, with an expected 2-year transplant-related mortality rate of 7.1%（95% *CI* 0%–21.1%）and an expected 2-year overall survival rate of 92.9%（95% *CI* 80.3%–100.0%）. Moreover, the expected 1-year and 2-year progression-free survival rates were 92.9%（95% *CI* 80.3%–100.0%）and 66.0%（95% *CI* 39.4%–100.0%）, respectively, and the 2-year cumulative incidence of relapse was 28.9%（95% *CI* 0%–56.7%）. Upfront allo-HSCT following complete remission after induced therapy and the presence of chronic GVHD might be favorable prognostic factors.

**Conclusion:**

allo-HSCT is an effective treatment for improving the prognosis of young patients with HRMM.

多发性骨髓瘤（multiple myeloma, MM）是一种克隆性浆细胞异常增殖的恶性疾病，约占血液系统恶性肿瘤的18％，中位发病年龄为69岁[1]。近年来，随着蛋白酶体抑制剂、免疫调节剂、抗CD38单抗、嵌合抗原受体T细胞（CAR-T）等新型治疗手段的涌现，MM患者的预后获得显著改善[2]。但MM疾病异质性强，个体间预后差异较大，国际骨髓瘤工作组（IMWG）指出部分具有特定细胞遗传学改变、较重疾病负荷、较差治疗反应等高危因素的MM患者中位生存期仍仅为3年左右[3]。

异基因造血干细胞移植（allo-HSCT）具有较强的移植物抗骨髓瘤（GVM）效应，因而被认定为MM可能的治愈方法[4]，但因其适应人群较高的移植相关死亡率（TRM）而倍受争议；有研究显示MM患者行allo-HSCT可能不会获益，亦有研究指出allo-HSCT可能提升MM患者尤其是年轻患者的长期生存率及治愈率[5]–[6]。《中国多发性骨髓瘤诊治指南（2022年修订）》[7]指出高危MM患者在有合适供者的情况下可考虑allo-HSCT。本研究回顾性分析了中国医学科学院血液病医院造血干细胞移植中心14例行allo-HSCT的年轻高危多发性骨髓瘤（HRMM）患者的临床资料，探讨allo-HSCT对年轻HRMM患者的疗效及预后影响。

## 病例与方法

一、病例

对2016年11月至2022年11月期间于中国医学科学院血液病医院造血干细胞移植中心接受allo-HSCT的14例MM患者的临床资料进行回顾性研究。纳入标准：①年龄≤50岁；②具有高危预后因素；③移植后随访≥100 d。MM的诊断标准均符合《中国多发性骨髓瘤诊治指南（2022年修订）》[7]，根据Durie-Salmon分期标准、第2版多发性骨髓瘤总生存期国际分期系统（R2-ISS）[8]、2016版IMWG指南[9]等进行分期及预后分层。本研究为研究者发起，符合伦理原则；所有患者均签署知情同意书。

二、预处理方案及移植物抗宿主病（GVHD）预防方案

14例患者全部接受清髓性预处理（MAC）；其中7例患者接受白消安联合美法仑及环磷酰胺方案，3例患者在应用白消安联合美法仑及环磷酰胺方案基础上加入氟达拉滨；另有3例患者所用方案在TBI联合环磷酰胺的基础上分别加用白消安、美法仑、氟达拉滨；此外，1例患者采用TBI联合美法仑方案。11例患者预处理方案中包含抗胸腺细胞球蛋白（ATG）。

7例患者行同胞供者全相合造血干细胞移植（MSD-HSCT），其中6例采用环孢素A（CsA）+短疗程甲氨蝶呤（MTX）方案预防GVHD，1例患者采用他克莫司（FK506）+短疗程MTX方案。5例行单倍体造血干细胞移植（haplo-HSCT），其中4例患者采用FK506+MMF+短疗程MTX方案，1例患者采用CsA+MMF+短疗程MTX方案。2例患者行无关供者造血干细胞移植（UD-HSCT），均采用FK506+MMF+短疗程MTX方案。

三、定义及疗效评估标准

MM疗效评价参考2016版IMWG指南[9]，包括严格意义的完全缓解（sCR）、完全缓解（CR）、非常好的部分缓解（VGPR）、部分缓解（PR）、疾病稳定（SD）和疾病进展（PD）等。高危细胞遗传学异常定义参照2016年IMWG高危多发性骨髓瘤治疗指南[3]，移植前治疗线数定义参照2015年美国血液学会指南[10]。根据《中国多发性骨髓瘤诊治指南（2022年修订）》[7]，本研究将移植后复发定义为获得CR疗效后复发。总生存（OS）期定义为从移植当日至死亡或末次随访的时间，无进展生存（PFS）期定义为从移植至MRD转阳或疾病进展或死亡或末次随访的时间。移植相关死亡（TRM）定义为allo-HSCT后发生的非复发/疾病进展导致的死亡。粒细胞植入定义为停用粒细胞集落刺激因子（G-CSF）后中性粒细胞计数（ANC）≥0.5×10^9^/L且持续3 d；血小板植入定义为PLT≥20×10^9^/L持续7 d且脱离血小板输注。

四、随访

通过查阅住院/门诊病历和电话随访方式获得患者生存资料及相关信息。主要观察指标为OS及PFS，次要观察指标为造血重建、急性GVHD、慢性GVHD、TRM等。随访截止时间为2023年7月1日，无法随访的患者将最后一次随访日期作为删失时间。移植后中位随访18.9（4.1～72.5）个月。

五、统计学处理

使用*R* 4.2.2软件进行统计学分析，Kaplan-Meier法进行生存分析计算生存率及中位生存时间，Log-rank法进行单因素分析，*P*<0.05为差异有统计学意义。

## 结果

一、患者临床特征及高危因素分析

14例患者中男7例，女7例；中位移植年龄为39.5（31～50）岁。IgG型6例，IgA型3例，IgD型2例，轻链型3例。Duria-Salmon分期：ⅡA期1例，ⅡB期1例，ⅢA期9例，ⅢB期3例；R2-ISS分期：Ⅱ期4例，Ⅲ期8例，Ⅳ期2例。14例患者均具高危因素，其中11例者具有高危细胞遗传学改变，4例患者初诊时诊断为原发性浆细胞白血病，1例患者初诊时合并髓外病变，具体见表1。

**表1:** 14例接受异基因造血干细胞移植高危多发性骨髓瘤患者的临床特征

例号	性别	年龄	疾病亚型	DS分期	R2-ISS分期	高危因素		初诊至移植时间（d）
						细胞遗传学异常	疾病临床状态	
1	女	43	IgA	ⅢA	Ⅳ	Del(17p)	/	389
2	男	32	κ轻链	ⅡB	Ⅲ	Del(13q)、t(4;14)	/	208
3	男	31	IgG	ⅢA	Ⅲ	gain(1q)、Del(17p)	/	379
4	男	37	IgD	ⅢB	Ⅲ	gain(1q)	/	280
5	女	34	IgD	ⅢB	Ⅲ	gain(1q)	浆细胞白血病	290
6	女	40	IgG	ⅢA	Ⅲ	gain(1q)、Del(13q)、t(11;14)	浆细胞白血病	569
7	女	44	IgG	ⅢA	Ⅲ	gain(1q)、Del(17p)	/	168
8	女	47	λ轻链	ⅡA	Ⅱ	/	髓外病变	353
9	男	50	IgG	ⅢA	Ⅳ	gain(1q)、Del(17p)	/	458
10	男	44	IgA	ⅢA	II	gain(1q)	/	238
11	男	32	κ轻链	ⅢA	Ⅲ	gain(1q)	/	217
12	男	42	IgG	ⅢA	Ⅱ	/	浆细胞白血病	227
13	女	39	IgA	ⅢA	Ⅲ	gain(1q)、t(4;14)	/	320
14	女	31	IgG	ⅢB	Ⅱ	/	浆细胞白血病	184

注 DS分期：Durie-Salmon分期；R2-ISS：第二版修订国际分期系统

二、治疗情况及疗效

患者初诊至移植中位时间为285（168～569）d，移植前中位治疗线数为2（1～6）线；14例患者均用过蛋白酶体抑制剂，12例（85.8％）患者用过来那度胺、沙利度胺等免疫调节剂，6例患者用过CD38单抗，另有2例（14.3％）难治患者有BMCA-CAR-T细胞治疗史，1例患者曾行一线auto-HSCT。移植前疾病状态：CR8例，VGPR 4例，PR 2例；移植前MRD阳性5例。具体见表2。

**表2:** 14例高危多发性骨髓瘤（HRMM）患者异基因造血干细胞移植前治疗情况

例号	既往治疗	治疗线数	移植时疾病状态	移植时MRD状态	移植前是否复发/难治
1	蛋白酶体抑制剂，免疫调节剂，抗CD38单抗	1	sCR	/	/
2	蛋白酶体抑制剂，免疫调节剂，抗CD38单抗	2	sCR	/	/
3	蛋白酶体抑制剂，免疫调节剂	2	PR	（+）	/
4	蛋白酶体抑制剂，免疫调节剂，抗CD38单抗	1	CR	（+）	MRD阴性后复发
5	蛋白酶体抑制剂，免疫调节剂，抗CD38单抗	2	sCR	/	/
6	蛋白酶体抑制剂，免疫调节剂，auto-HSCT	4	sCR	（+）	MRD阴性后复发
7	蛋白酶体抑制剂，免疫调节剂	1	VGPR	（+）	/
8	蛋白酶体抑制剂，抗CD38单抗	1	sCR	/	/
9	蛋白酶体抑制剂，免疫调节剂，抗CD38单抗，CAR-T	6	PR	/	难治
10	蛋白酶体抑制剂，免疫调节剂	2	VGPR	（+）	/
11	蛋白酶体抑制剂	1	sCR	/	/
12	蛋白酶体抑制剂，免疫调节剂	1	VGPR	/	/
13	蛋白酶体抑制剂，免疫调节剂，抗CD38单抗，CAR-T	4	sCR	/	难治
14	蛋白酶体抑制剂，免疫调节剂，抗CD38单抗	4	VGPR	/	/

注 sCR：严格意义的完全缓解；CR：完全缓解；VGPR：非常好的部分缓解；PR：部分缓解；MRD：微小残留病。auto-HSCT：自体造血干细胞移植；CAR-T：嵌合抗原受体T细胞疗法

9例（64.3％）患者为诱导治疗后一线行allo-HSCT，5例（35.7％）患者因为难治/复发而采用allo-HSCT挽救治疗。7例患者行MSD-HSCT，5例行haplo-HSCT，2例行URD-HSCT。移植后3个月疗效评估，所有患者均为CR。具体见表3。

**表3:** 14例高危多发性骨髓瘤（HRMM）患者异基因造血干细胞移植情况及随访结果

例号	供者类型	预处理方案	GVHD预防方案	ATG使用	MNC输注量（×10^8^/kg）	CD34^+^细胞输注量（×10^6^/kg）	移植后最佳疗效	中性粒细胞植入时间（d）	血小板植入时间（d）	急性GVHD	慢性GVHD	血液学复发	存活	随访时间（月）
1	haplo	Mel+Flu+Bu+CTX	FK506+MMF+MTX	+	10.88	5.33	sCR	11	13	–	否	–	+	8.0
2	MSD	Mel+Flu+Bu+CTX	FK506+MTX	–	8.00	6.56	sCR	12	15	–	重度广泛	–	+	9.6
3	MSD	Mel+Bu+CTX	FK506+MTX	–	14.21	4.08	sCR	11	12	Ⅲ度	轻度局限	–	+	22.4
4	haplo	Mel+Bu+CTX	FK506+MMF+MTX	+	12.50	2.76	sCR	10	103	Ⅲ度	否	MRD（+）^a^	+	21.3
5	haplo	Mel+Flu+Bu+CTX	FK506+MMF+MTX	+	10.91	3.51	sCR	10	10	–	轻度局限	–	+	9.5
6	MSD	TBI+CTX+Flu	FK506+MTX	+	9.77	2.83	sCR	11	22	–	否	+	+	20.7
7	MSD	Mel+Bu+CTX	FK506+MTX	+	12.34	5.89	sCR	10	9	–	否	–	+	14.5
8	MSD	Mel+Bu+CTX	FK506+MTX	+	11.69	2.17	sCR	11	11	–	重度广泛	–	+	24.2
9	UD	Mel+Bu+CTX	FK506+MMF+MTX	+	7.00	7.64	sCR	11	12	Ⅱ度	轻度局限	+	+	9.2
10	MSD	Mel+Bu+CTX	FK506+MTX	+	10.00	2.40	sCR	10	13	–	轻度局限	–	+	19.5
11	UD	TBI+Mel	FK506+MMF+MTX	+	5.80	3.48	sCR	11	12	–	否	–	+	72.5
12	haplo	TBI+Mel+CTX	CsA+MMF	+	13.87	5.85	sCR	10	11	–	否	–	+	18.9
13	haplo	Mel+Bu+CTX	FK506+MTX+MMF	+	12.39	2.55	sCR	14	16	Ⅲ度	否	–	–	4.1
14	MSD	TBI+Bu+CTX	CsA+MTX	–	7.69	3.61	sCR	14	13	Ⅱ度	否	MRD（+）^a^	+	4.1

注 GVHD：移植物抗宿主病；ATG：抗胸腺细胞球蛋白；Bu：白消安；CsA：环孢素A；CTX：环磷酰胺；FK506：他克莫司；Flu：氟达拉滨；haplo：单倍体造血干细胞移植；MSD：同胞全相合造血干细胞移植；UD：无关供者造血干细胞移植；Mel：美法仑；MMF：霉酚酸酯；MTX：甲氨蝶呤；MNC：单个核细胞；TBI：全身放射治疗；MRD：微小残留病；sCR：严格意义的完全缓解；^a^ 完全缓解后MRD转阳，无血液学复发

三、造血重建

14例患者CD34^+^细胞中位输注量为3.56（2.17～7.64）×10^6^/kg。中位中性粒细胞、血小板植入时间分别为11（10～14）d、13（9～103）d，13例（92.9％）患者于移植后28 d内血小板植入，另1例患者血小板植入时间为移植后103 d。

四、GVHD

5例（35.7％）患者发生Ⅱ～Ⅳ度急性GVHD，1例单独表现为皮肤Ⅲ级，1例单独表现为肠道1级，另有1例Ⅲ度急性GVHD患者表现为肝脏2级合并肠道1级，其余2例Ⅲ度急性GVHD患者表现为严重腹泻、便血伴腹痛。此外2例患者发生中重度慢性GVHD，均以黄疸、肝酶升高、口腔溃疡、皮肤增厚、眼干等为主要表现。

五、生存情况

随访时间至2023年7月1日，14例患者的中位随访时间为18.9（4.1～72.5）月。移植后2年TRM为7.1％（95％ *CI* 0％～21.1％），1例患者于移植后5个月因合并4级肠道急性GVHD、移植相关血栓性微血管病（TA-TMA）、腺病毒血流感染而死亡，其余13例患者均存活，最长随访时间为6年，预期2年OS率为92.9％（95％ *CI* 80.3％～100.0％），累积复发率（CIR）为28.9％（95％ *CI* 0％～56.7％），其中2例患者分别于移植后12.2、20.7个月出现CR后血液学复发；予以供者淋巴细胞输注后分别评价为PR及SD，目前均存活，预期1、2年PFS率分别为92.9％（95％*CI* 80.3％～100.0％）、66.0％（95％ *CI* 39.4％～100.0％）。另有2例患者移植后出现MRD转阳，其中1例患者移植后4.1个月MRD转阳（失访），另1例患者移植后21个月MRD阳性，予以泊马度胺4 mg/d治疗后MRD转阴。生存曲线见图1。

**图1:**
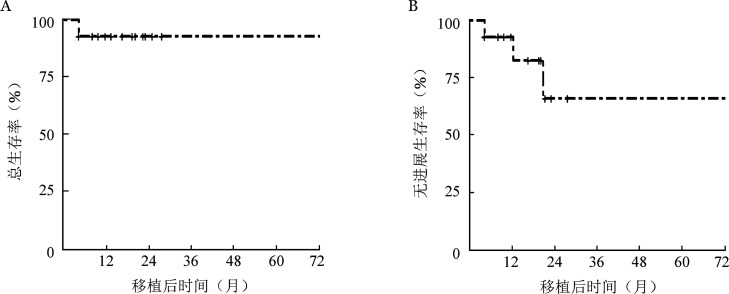
14例高危多发性骨髓瘤（HRMM）患者异基因造血干细胞移植后总生存曲线（A）和无进展生存曲线（B）

## 讨论

allo-HSCT具有GVM效应，可改善MM患者预后，甚至是该病的唯一治愈方法[11]。新药时代仍有部分HRMM患者对多种药物治疗反应较差、复发率高，预后不佳。但allo-HSCT治疗MM有较高的TRM发生率，因此倍受争议。多项研究表明，随着治疗手段及支持治疗的进步，近年来allo-HSCT治疗MM患者TRM较前大幅降低[12]–[16]。本研究拟探究支持治疗手段进步及供者选择范围扩大的情况下，allo-HSCT对年轻HRMM的疗效。

为最大程度发挥allo-HSCT的GVM效应、避免MAC方案的毒性并兼顾减低移植前肿瘤负荷，国外多个中心尝试auto-HSCT序贯减低强度预处理（RIC）allo-HSCT治疗MM，TRM降至11％～16％[17]–[19]。Beaussant等[20]比较了采用MAC（49例）和RIC（397例）allo-HSCT治疗MM患者的临床资料，显示在年轻、无合并症患者中MAC组与RIC组TRM未见明显差异（22.4％对24.6％），且MAC方案可能对OS、PFS有益。本组患者年龄均≤50岁且均采用MAC方案，移植后2年TRM仅为7.1％，表明在充分支持治疗支持下年轻患者采用MAC方案进行allo-HSCT可维持TRM于较低水平。

关注新药达雷妥尤单抗的POLLUX研究及探究卡非佐米疗效的ENDEAVO试验[22]均显示新药改善了HRMM患者的预后，但未完全克服高危因素的负面影响。Yin等[11]的荟萃研究（纳入8 698例allo-HSCT患者）表明，HRMM患者与标危MM患者具有相似的移植后OS及PFS。Greil等[6]研究显示，MM患者allo-HSCT后10年生存曲线可达平台期，10年OS、PFS率分别为26.1％、20.1％，这表明部分特定亚组的患者已经可能被治愈。本研究全部患者年龄均≤50岁，均表现为高危生物学特性，移植后患者ORR为100.0％，2年OS率为92.9％（95％ *CI* 80.3％～100.0％），2年CIR为28.9％（95％ *CI* 0％～56.7％），2年PFS率为66.0％（95％ *CI* 39.4％～100.0％），2年OS率高于既往研究[23]–[25]，2年PFS率则与文献[26]结果相仿，我们推测这与浆细胞白血病患者占比较高相关。近期Schmidt等[27]研究显示，MM患者行allo-HSCT后的中位OS期为1.7年，2年OS率低于50％；该研究中大部分患者在allo-HSCT前处于复发/难治状态，超过75％的患者移植前经历过3线及以上治疗，仅18％的患者移植前达CR，其较高的1年TRM（23.5％）及较低的OS率可能与移植前疾病状态相关。可见，allo-HSCT可显著改善MM患者，尤其移植前治疗线数相对较少患者的预后，且对OS的改善可能更为显著。

本研究中9例诱导治疗后一线行allo-HSCT的患者目前均为无病存活状态（最长已达6年），2例复发及2例MRD转阳的患者均已allo-HSCT作为挽救性治疗手段。目前，国内外各指南对allo-HSCT在MM中的应用时机仍未形成共识。据EBMT慢性恶性肿瘤工作组报告，欧洲部分国家推荐高危患者诱导后行一线allo-HSCT，也有国家推荐auto-HSCT序贯allo-HSCT，还有国家推荐复发/难治后挽救性移植[4]。Green等[28]采用auto-HSCT序贯allo-HSCT方案治疗24例新诊断HRMM及7例复发/难治MM患者，结果显示新诊断HRMM组2年OS率、PFS率分别为75％、71％，复发/难治组2年OS率、PFS率分别为43％、14％。另有研究表明相较于双次auto-HSCT，auto-HSCT序贯allo-HSCT患者移植复发后生存率明显提高[29]。我们认为对于年轻HRMM患者，其疾病复发/进展的风险可能大于移植相关风险，多线药物治疗亦可削弱患者身体耐受能力，且allo-HSCT显著改善患者移植复发后OS率，allo-HSCT应当作为HRMM患者初始诱导获得CR后首选一线治疗方案；其作为复发、难治HRMM患者挽救性治疗方案的获益仍有待进一步研究。

本研究中6例患者发生慢性GVHD，无患者因慢性GVHD死亡，1例患者复发；未发生慢性GVHD的8例患者中1例复发、2例MRD转阳。这与既往多项研究显示较高的慢性GVHD发生率可能是allo-HSCT后良好预后因素的结论相似。慢性GVHD及患者结局之间的关系可能更加复杂，未来仍需深入研究[29]。此外，kawamura等[30]研究显示，移植前疗效是否达到VGPR是影响allo-HSCT疗效的独立预测因素。本研究中移植前达CR的8例患者中1例死亡，1例复发；未达CR的6例患者中1例复发，CR患者与未达CR患者预后未见明显差异，但本研究研究例数较少，HRMM诱导治疗后未达CR患者能否从allo-HSCT中获取与诱导治疗后达CR患者相似的治疗受益这一结论仍需更大样本量研究加以证实。

多项研究显示，auto-HSCT序贯来那度胺维持治疗可改善MM患者OS及PFS[31]。本研究中采用移植后维持治疗的病例较少，因此难以明确移植后不同维持治疗方式对患者预后和GVHD发生率的影响。

综上，本研究结果初步显示，≤50岁的HRMM患者在诱导治疗后桥接allo-HSCT可进一步提高疗效。本研究为单中心回顾性研究，样本量较小，随访时间较短，以上结论尚需开展前瞻性多中心研究探索加以验证。
